# Connexin-43 Gap Junctions Are Responsible for the Hypothalamic Tanycyte-Coupled Network

**DOI:** 10.3389/fncel.2018.00406

**Published:** 2018-11-26

**Authors:** Antonia Recabal, Roberto Elizondo-Vega, Camille Philippot, Magdiel Salgado, Sergio López, Alejandra Palma, Estefanía Tarifeño-Saldivia, Aline Timmermann, Gerald Seifert, Teresa Caprile, Christian Steinhäuser, María Angeles García-Robles

**Affiliations:** ^1^Department of Cellular Biology, Faculty of Biological Sciences, University of Concepcion Concepcion, Chile; ^2^Institute of Cellular Neurosciences, Medical Faculty, University of Bonn Bonn, Germany; ^3^Department of Biochemistry and Molecular Biology, Faculty of Biological Sciences, University of Concepcion Concepcion, Chile

**Keywords:** hypothalamus, gap junctions, tanycytes, astrocytes, proliferation

## Abstract

Tanycytes are hypothalamic radial glia-like cells that form the basal wall of the third ventricle (3V) where they sense glucose and modulate neighboring neuronal activity to control feeding behavior. This role requires the coupling of hypothalamic cells since transient decreased hypothalamic Cx43 expression inhibits the increase of brain glucose-induced insulin secretion. Tanycytes have been postulated as possible hypothalamic neuronal precursors due to their privileged position in the hypothalamus that allows them to detect mitogenic signals and because they share the markers and characteristics of neuronal precursors located in other neurogenic niches, including the formation of coupled networks through connexins. Using wild-type (WT), Cx30^−/–^ and Cx30^−/–^, Cx43^fl/fl^:glial fibrillary acidic protein (GFAP)-Cre (double knockout, dKO) mouse lines, we demonstrated that tanycytes are highly coupled to each other and also give rise to a panglial network specifically through Cx43. Using the human GFAP (hGFAP)-enhanced green fluorescent protein (EGFP) transgenic mouse line, we provided evidence that the main parenchymal-coupled cells were astrocytes. In addition, electrophysiological parameters, such as membrane resistance, were altered when Cx43 was genetically absent or pharmacologically inhibited. Finally, in the dKO mouse line, we detected a significant decrease in the number of hypothalamic proliferative parenchymal cells. Our results demonstrate the importance of Cx43 in tanycyte homotypic and panglial coupling and show that Cx43 function influences the proliferative potential of hypothalamic cells.

## Introduction

Tanycytes are radial glia-like cells that surround the lateral walls of the infundibular recess, with their cell bodies facing the third ventricle (3V) and long processes extending deep into the parenchyma. Tanycytes are classified into the following four main groups on the basis of differences in their localization and gene expression: α1, α2 (Robins et al., 2013), β1 and β2 (Rodríguez et al., 2005). α1-, α2- and β-tanycytes are located in the lateral walls of the 3V. While α2- and β1-tanycytes contact anorexigenic and orexigenic neurons of the arcuate nucleus through their extensive processes, α1 do so with the neurons residing in the ventromedial nucleus. β2-tanycytes cover the floor of the 3V and present tight junctions that form the cerebrospinal fluid (CSF)-median eminence (ME) barrier.

It has been proposed that tanycytes modulate the activity of hypothalamic neurons (García et al., 2003; Elizondo-Vega et al., 2015, 2016) given that they have access to hormones in the circulating blood (Langlet et al., 2013; Balland et al., 2014; Collden et al., 2015) and also express the molecular machinery that permits glucosensing (García et al., 2003; Córtés-Campos et al., 2011; Orellana et al., 2012). Moreover, tanycytes may act as adult hypothalamic neuronal precursor cells (NPCs; Lee and Blackshaw, 2012; Haan et al., 2013; Robins et al., 2013; Jourdon et al., 2016) because they express radial glia markers, are capable of forming neurospheres (Robins et al., 2013), and can proliferate and differentiate into glia and functionally leptin-activating neurons. Neuronal degeneration (Yulyaningsih et al., 2017), environmental changes in the diet (Lee and Blackshaw, 2012; Gouazé et al., 2013), and intracerebroventricular administration of intrinsic factors are known to accelerate the low rate of basal hypothalamic cell division (Pencea et al., 2001; Kokoeva et al., 2005; Pérez-Martín et al., 2010; Yulyaningsih et al., 2017) in order to restore the energy balance. However, the mechanisms underlying the proliferation and synchronization of the cell cycle in tanycytes remain to be elucidated.

In radial glia cells, the existence of a coupled network may play a role in the regulation and synchronization of the cell cycle through calcium waves (Bittman et al., 1997), occurring spontaneously and requiring the purinergic P2Y1 receptor and intracellular inositol trisphosphate (IP_3_)-mediated calcium release (Weissman et al., 2004). NPCs are characterized by a robust coupling and high expression of Cx43 even in different brain regions and developmental stages, such as early postnatal NPCs (Freitas et al., 2012), those from the adult subventricular zone (SVZ; Lacar et al., 2011), neurospheres originating from the adult SVZ (Talaverón et al., 2015), radial glia-like cells in the subgranular zone (SGZ) of the adult dentate gyrus (Kunze et al., 2009), and proliferating neurogenic precursors of the spinal cord (Russo et al., 2008).

Tanycytes express Cx43 as a part of their glucosensing mechanism, which includes glucose transporter 2 (GLUT2; Barahona et al., 2018) and glucokinase (GK), which both are expressed by α1-, α2- and β1-tanycytes. Moreover, glucose triggers ATP release that activates P2Y1 *in vitro* (Orellana et al., 2012) and generates calcium waves in tanycytes in acute slices (Frayling et al., 2011). Tanycyte sub-populations show different responses to glucose analogs, and it remains to be shown whether Cx43-hemichannels contribute to these responses. Nevertheless, it is unknown whether calcium waves in tanycytes are spread intracellularly through gap junctions or extracellularly by a diffusible signal. A previous study showed that tanycytes form a coupled “syncytium” (Jarvis and Andrew, 1988; Szilvásy-Szabó et al., 2017), which is uncoupled using carbenoxolone (CBX), a general blocker of gap junctions. However, the connexins involved in tanycyte coupling remain undescribed.

Here, using wild-type (WT), Cx30^−/–^ (knockout, KO), and Cx30^−/–^, Cx43^fl/fl^:glial fibrillary acidic protein (GFAP)-Cre (double KO, dKO) mouse lines (Wallraff et al., 2006; Zhang et al., 2013), we demonstrated that tanycytes are highly coupled to each other and also give rise to a panglial network specifically through Cx43. Using the human GFAP (hGFAP)-EGFP transgenic mouse line, we provide evidence that some parenchymal-coupled cells were astrocytes. Additionally, by immunohistochemistry, we detected tanycyte-oligodendrocyte coupling. We also showed that their electrophysiological properties, such as membrane resistance, were altered when Cx43 was genetically absent or pharmacologically inhibited. Finally, basal hypothalamic cell proliferation was altered when connexin 43 expression was suppressed.

## Materials and Methods

### Ethics Statement

All studies performed using rats were reviewed and approved by the Animal Ethics Committee of the Chile’s National Commission for Scientific and Technological Research (CONICYT, protocol for projects # 1180871). All animal work was approved by the appropriate Ethics and Animal Care and Use Committee of the Universidad de Concepcion, Chile. Animals were treated in compliance with the U.S. National Institutes of Health guidelines for animal care and use. Adult Sprague-Dawley rats (200–280 g) were housed in a 12-h light/dark cycle with food and water *ad libitum*. Adult C57BL6/J (Charles River, Wilmington, DE, USA), Cx30^−/–^ (Cx30 KO); Cx30^−/–^, Cx43^fl/fl^:hGFAP-Cre (dKO; Zhang et al., 2013) and hGFAP-EGFP (Nolte et al., 2001) mice of either sex were investigated at postnatal (p) days 38–119 if not indicated otherwise. Mice were kept under standard housing conditions (12 h light/dark cycle, water and food *ad libitum*).

### Tanycyte Primary Cultures

Cultures of hypothalamic tanycytes were obtained following a method described previously (García et al., 2003; Orellana et al., 2012). Rats at p1 were rapidly decapitated, their brains were removed and the region close to the ventricular region was dissected in a cold environment. Samples were incubated with 0.25% trypsin-0.2% EDTA (w/v) for 20 min at 37°C and transferred to MEM (Invitrogen, Life Technologies, Darmstadt, Germany) supplemented with 10% FBS, 2 mM L-glutamine, 100 U/mL penicillin and 100 μg/mL streptomycin (Thermo Fisher Scientific, Auckland, New Zealand). Sections were disaggregated, and cells were seeded on T25 dishes coated with 0.2 mg/mL poly-L-lysine (Sigma-Aldrich, Steinheim, Germany) at a density of over 3 × 10^6^ cells per dish. Cells were cultured in the same dish for 2 weeks, and the media was renewed every 2 days.

For subsequent experimental procedures, tanycytes were washed twice in 0.1 M phosphate buffer solution (PBS), pH 7.4 and treated with 0.25% trypsin-0.2% EDTA for 3 min at 37°C. Cells were disaggregated and reseeded in 6-well plates previously coated with poly-L-lysine at an approximately cell density of 5 × 10^5^ cells per dish. Cells were used up to the first passage.

### Real-Time Quantitative Reverse Transcription-Polymerase Chain Reaction (Real-Time RT-qPCR)

The brain of each rat was removed, and hypothalamic areas were isolated and further dissected. Total RNA from tanycyte primary cultures and hypothalamic samples was isolated using TRIZOL (Thermo Fisher Scientific, Waltham, MA, USA) and treated with DNase I (Fermentas International, Burlington, ON, Canada). Reverse transcription polymerase chain reaction (RT-PCR) was performed according to the manufacturer’s protocol (Fermentas International) using 2 μg of RNA and 20 μl reaction volume containing 10 mM Tris-HCl (pH 8.3), 50 mM KCl, 5 mM MgCl_2_, 20 U RNase inhibitor, 1 mM dNTPs, 2.5 μM of oligo d(T) primers, and 50 units of MuLV reverse transcriptase (New England Biolabs, Ipswich, MA, USA) for 60 min at 42°C followed by 10 min at 70°C. Parallel reactions were performed in the absence of reverse transcriptase to control for the presence of genomic DNA. RT-qPCR reactions were prepared with a Brilliant II SYBR Green qPCR Master Mix kit (Agilent Technologies, Santa Clara, CA, USA) in a final volume of 20 μL containing 2 μL cDNA and the following sets of primers (500 nM each): Cx26, sense 5′-TTG CTC AGG GAA GTC CAA AAG ACC-3′ and antisense 5′-TGG GCC TTT GTT TGG GAG CTT T-3′ (expected product of 231 bp); Cx30, sense 5′-GTG AAC AAG CAC TCG ACC AGC ATA-3′ and antisense 5′-GCG GAT AAA CTT TCG GGC AGT TTC-3′ (expected product of 288 bp); Cx43, sense 5′-TGG GTA CAA GCT GGT TAC TGG TGA-3′ and antisense 5′-TGG CTA ATG GCT GGA GTT CAT GTC-3′ (expected product of 221 bp); and cyclophilin, sense 5′-ATA ATG GCA CTG GTG GCA AGT C-3′ and antisense 5′-ATT CCT GGA CCC AAA ACG CTC C-3′ (expected product of 239 bp); Cx45, sense 5′-AAA GAG CAG AGC CAA CCA-3′ and antisense 5′-CCA AAC CCT AAG TGA AGC-3′ (expected product of 311 bp); Px1, sense 5′-TTC TTC CCC TAC ATC CTG CT-3′ and antisense 5′-GGT CCA TCT CTC AGG TCC AA-3′ (expected product of 185 bp), Px2, sense 5′-TGG ACA TCG TAT TGC TCT GC-3′ and antisense 5′-CCA CGT TGT CGT ACA TGA GG-3′ (expected product of 258 bp). Each reaction mixture was incubated at 95°C for 5 min followed by 40 cycles at 95°C for 30 s, 60°C for 30 s and 72°C for 30 s and a final extension of 7 min at 72°C. The efficiency of the primer pairs (Cx26, Cx30 and Cx43) were 101.5%, 104.1% and 121.4%, respectively. The relative expression of Cx26, Cx30 and Cx43 was calculated by the ΔCT method using cyclophilin as the housekeeping control gene.

### RNA Sequencing Analysis for Connexin Identification

RNAseq analysis was performed in tanycyte primary cultures maintained in 2 mM and 15 mM glucose to evaluate the transcriptional effects of glucose at two different concentrations. Both concentrations have been detected in the CSF during hypoglycemia and hyperglycemia conditions, respectively (Salgado et al., 2014). Primary cultures were maintained at 2 mM glucose for 24 h, after which glucose concentrations were increased to 15 mM for 2 h. At the end of these periods, total RNA was extracted to prepare the Truseq Illumina libraries. Sequencing was performed to obtain single-ends with a coverage of 90 million reads (50 bp) per library. Sequences were trimmed in order to remove adaptors and low-quality bases. Trimmed reads were mapped into the genome (Ensembl genome version 6.0.81, ensemble.org) using Tophat v.2.0.9 (Trapnell et al., 2012). Gene expression was measured from the mapped reads using HTseq-count (Anders et al., 2015). The RNAseq dataset generated in this study is available at the European nucleotide archive (ENA), access number PRJEB28405.

### Antibodies and Immunological Assays

For streptavidin staining, 200 μm hypothalamic slices were washed three times for 10 min in 0.1 M PBS (17 mM Na_2_HPO_4_, 83 mM NaH_2_PO_4_· 2H_2_O, 15 mM NaCl, pH 7.4) and then blocked with a solution containing 10% normal goat serum (Chemicon Temecula, CA, USA) and 2% Triton X-100 in PBS for 2 h at room temperature. Slices were incubated overnight at 4°C with the primary antibodies. Then, the primary antibodies were washed three times for 10 min in 0.1 M PBS and incubated for 2 h at room temperature with the secondary antibodies at a 1:500 dilution ratio. Both primary and secondary antibodies were diluted in a solution containing 2% normal goat serum and 0.1% Triton X-100 in PBS. The slices were placed on a slide and mounted with Aqua Poly-mount (Polysciences Europe, Eppelheim, Germany). For other stainings, 40 μm hypothalamic slices were incubated in Tris phosphate buffer instead of PBS. For bromodeoxyuridine (BrdU) immunohistochemistry, an additional step of DNA denaturation was performed by incubating the slices with 1 M HCl at 45°C for 30 min before blocking. The acid was neutralized by rinsing the sections three times for 10 min with Tris phosphate buffer on a shaker. The following primary and secondary antibodies were used: streptavidin conjugated to Alexa 647 (1:600, Invitrogen, Molecular Probes, Darmstadt, Germany), rabbit anti-GFAP (1:200, DAKO, Campintene, CA, USA), chicken anti-GFAP (1:500, Synaptic Systems, Göttingen, Germany), mouse anti-GSTpi (1:200, BD Transduction Laboratories, San Jose, CA, USA), chicken anti-GFP (1:500, Abcam, Cambridge, UK), rabbit anti-connexin43 (1:200, Sigma Aldrich, Steinheim, Germany) and sheep anti-BrdU (1:500, Abcam). The following secondary antibodies were used at a dilution of 1:500: goat anti-mouse Alexa 488 (Invitrogen), goat anti-mouse Alexa 594 (Invitrogen), goat anti-rabbit Alexa 488 (Invitrogen), goat anti-rabbit Alexa 594 (Invitrogen), goat anti-rabbit Alexa 647 (Invitrogen, Molecular Probes), goat anti-rabbit Alexa 647 (Invitrogen), goat anti-chicken Alexa 488 (Invitrogen), goat anti-chicken Alexa 549 (Life Technologies) and goat anti-sheep Alexa 488 (Abcam). Images were subjected to background subtraction using ImageJ software.

### Preparation of Slices

Mice of both sexes and aged P39–P122 days (P39–122) were anesthetized with isoflurane (Abbott, Wiesbaden, Germany) and killed by decapitation. Brains were removed and coronal sections were performed using the rostral and caudal borders of the Willis polygon as reference. The sections were immersed in ice cold artificial CSF (aCSF) containing 1.25 mM NaH_2_PO_4_, 87 mM NaCl, 2.5 mM KCl, 7 mM MgCl_2_, 0.5 mM CaCl_2_, 25 mM glucose, 25 mM NaHCO_3_ and 50 mM sucrose (osmolarity of 336 mOsm) equilibrated with 95% O_2_ and 5% CO_2_ to a pH 7.4. Slices of 200 μm thickness were prepared using a vibratome (Leica VT 1200 S, Nussloch, GmbH) at a speed of 0.12 mm/s. Slices were transferred to the same equilibrated solution at 35°C for 20 min.

### Electrophysiology

Slices were stored in aCSF without sucrose containing 1.25 mM NaH_2_PO_4_, 126 mM NaCl, 3 mM KCl, 2 mM MgCl_2_, 2 mM CaCl_2_, 10 mM glucose and 26 mM NaHCO_3_ (305–315 mOsm) equilibrated with 95% O_2_ and 5% CO_2_ to pH 7.4 at room temperature. Whole-cell recordings were obtained using an EPC7 amplifier D-6100 (HEKA, Lambrecht/Pfalz, Germany) and borosilicate capillaries pipettes (GB150F-10, Science Products GmbH, Hofheim, Germany), which had resistances of 2–5 MΩ. Pipettes were fabricated with a puller (DMZ, Zeitz-Instruments, Germany) and were filled with a standard intracellular solution (130 mM K-gluconate, 1 mM MgCl_2_, 3 mM Na_2_-ATP, 20 mM HEPES and 10 mM EGTA, pH 7.2). For intracellular labeling, 0.5% biocytin (Nε-biotinyl-L-lysine; Sigma) and in some cases 300 μM dextran (Texas Red 3000 MW Lysine Fixable, Molecular Probes) were added to the pipette solution. Voltages were corrected for liquid junction potential. Series resistance was compensated for (up to 20%). Recordings were sampled at 30 kHz, filtered at 10 kHz, and monitored with TIDA software (HEKA, Lambrecht/Pfalz, Germany). Visual control of tanycytes was achieved with a microscope (FN-S2N, Nikon, Japan) with a 60× objective. Due to the central localization of the 3V, only one cell was recorded per individual slice. Tanycytes were filled for 20 min and cells were considered for evaluation if the membrane potential remained below −65 mV and −55 mV in WT and dKO slices, respectively. The access resistance was below 20 MΩ. After filling, slices were fixed in 4% paraformaldehyde (PFA) in 0.1 M PBS (17 mM Na_2_HPO_4_, 83 mM NaH_2_PO_4_·2H_2_O, 15 mM NaCl, pH 7.4) at 4°C overnight.

### Bromodeoxyuridine (BrdU) Injections in Mice

WT and dKO mice (39–50 days-old and of both sexes) were injected intraperitoneally with 100 μL of 50 mg/kg BrdU (Sigma Aldrich) once a day for 6 days (4 days of injection, 2 days of rest and 2 days of injection at 11:00 a.m.). BrdU was first dissolved in sterile saline (NaCl 0.9% p/v, pH 7.3), and a stock solution was stored at −20°C. The animals were left for another week without BrdU incorporation. In the second week following the first injection, mice were anesthetized with a mix of Ketamine-Xylazine (150/15 mg/kg) and vascularly perfused with 30 mL of ice cold 0.1 M PBS (17 mM Na_2_HPO_4_, 83 mM NaH_2_PO_4_·2H_2_O, 15 mM NaCl, pH 7.4) followed by 30 mL of 4% PFA dissolved in PBS. For PBS and PFA, the speed of perfusion was 300 mL/h controlled by a perfusor (B. Braun Melsungen AG, Germany) and by a syringe pump (model SP2201Z, WPI, Germany). The brains were removed and kept in 4% PFA at 4°C overnight. Hypothalamic tissues were dissected, and slices of 40-μm thickness were obtained using a vibratome (Leica VT 1200 S, Nussloch, GmbH) at a speed of 0.16 mm/s for subsequent immunohistochemistry analysis. The slices were preserved in 0.1 M PBS, pH 7.4 at 4°C for up to 2 weeks. For longer storage, sodium azide 0.01% was added.

### Cell Counts and Statistical Analysis

Streptavidin-labeled tanycytes were counted from a single focal plane selected as the one with maximum signal (each optical plane 1–2 μm). The number of streptavidin-labeled parenchymal cells was quantified using the ROI manager option in the ImageJ-software through multiple focal planes within the sections. Two investigators counted the number of whole Hoechst-positive nuclei, and the value was calculated as the average of both. We used at least 12 slices and four animals per genotype/condition, and the analyzed values were calculated as the means over each animal. Significant differences were determined using Student’s *t*-tests or one-way ANOVA with Bonferroni multiple comparison tests if not indicating otherwise. *p* < 0.05 was defined as significant using GraphPad Prism software.

## Results

### Cx43 Is the Most Abundant Connexin Expressed in Rat Tanycytes

**Figure 1:**
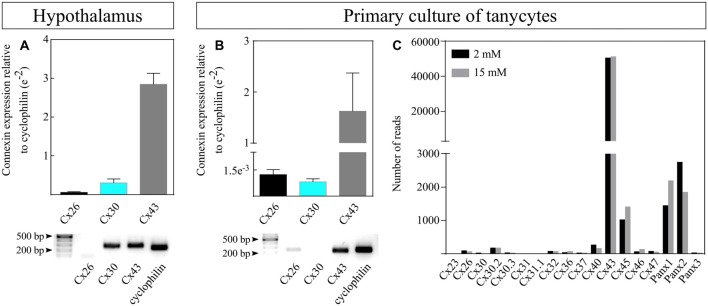
Cx43 is the most abundant connexin expressed in rat tanycytes. Expression of connexin Cx26, Cx30 and Cx43 was measured by reverse transcription-quantitative polymerase chain reaction (RT-qPCR) in **(A)** whole rat hypothalamic tissues (*N* = 3 animals) and **(B)** primary cultures of rat tanycytes (P1, *N* = 3 independent primary cultures). Transcriptional expression was normalized to the cyclophilin housekeeping gene. Representative electrophoresis of the RT-qPCR products is shown (bottom panels), although the blot for Cx30 was not detectable in the gel e = EXP. **(C)** Expression of connexins and pannexins (Px) obtained by RNAseq in primary cultures of rat tanycytes exposed to 2 and 15 mM glucose.

Previous immunocytochemistry and immunohistochemistry analyses have shown that Cx43 is expressed in tanycytes (Orellana et al., 2012; Szilvásy-Szabó et al., 2017). Here, we aimed to compare the expression of Cx43 with Cx26 and Cx30, which may be involved in the regulation of NPCs in other brain areas (Bittman and LoTurco, 1999; Liebmann et al., 2013) and are also expressed by astrocytes (Nagy and Rash, 2000). Through RT-qPCR, we confirmed the high expression of Cx43 in rat adult hypothalamus (Figure 1A) and primary cultures of rat tanycytes (Figure 1B). As shown in Figure 1A, Cx43 mRNA was 50 times more abundant than Cx26 and nine times higher than Cx30 in the hypothalamus (dark gray bar) while it was 1,300 times more abundant than Cx26 and almost 2,000 times higher than Cx30 in tanycyte cultures (Figure 1B, dark gray bar). Similar results were obtained in the electrophoretic assay shown in Figures 1A,B (lower panels), suggesting a specific function of Cx43 in this cell type. Moreover, RNA-seq data from primary cultured tanycytes exposed to 2 mM and 15 mM glucose, which correspond to hypoglycemic or hyperglycemic conditions respectively, confirmed that Cx43 is the main isoform expressed (Figure 1C) in this cell type. Additionally to Cx43 expression Px1-transcript is highly expressed in tanycytes cultures, Cx45 and Px2 are expressed in tanycytes albeit to a lower degree compared with Cx43 (Supplementary Figure S1). At the protein level, Cx45 is localized in all tanycyte subpopulations colocalizing with vimentin, mainly in β-tanycytes.

### Absence of Hypothalamic Cx43 Expression in dKO Mice

**Figure 2:**
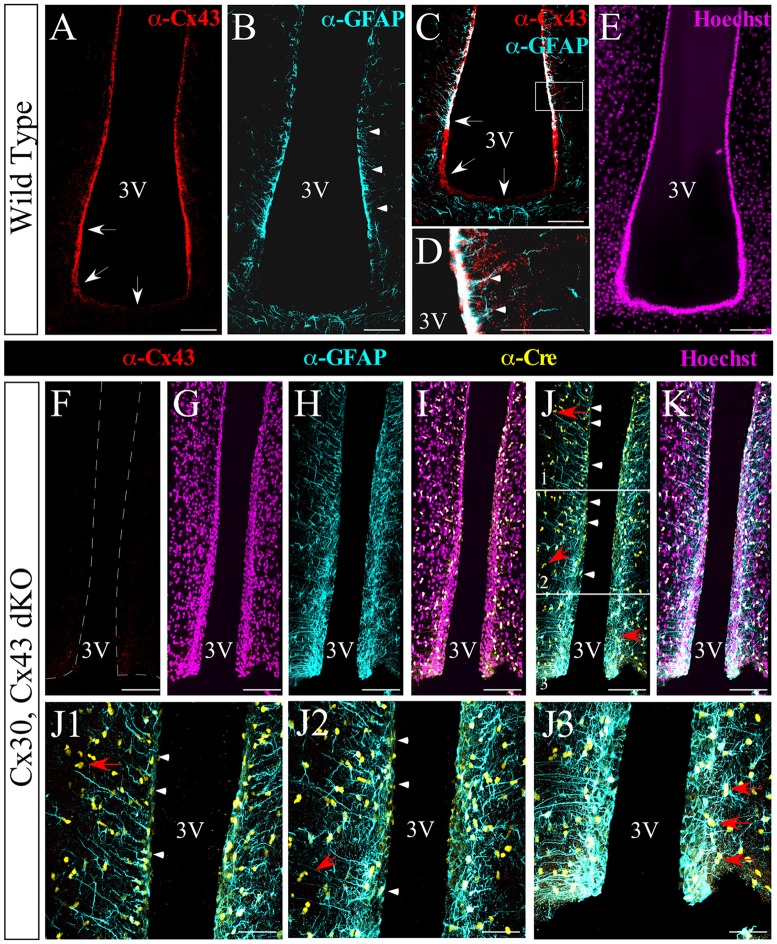
Absence of hypothalamic Cx43 expression in double knockout (dKO) mice. Immunohistochemical analysis of frontal hypothalamic slices obtained from wild-type (WT; **A–E**) and dKO **(F–J3)** mice. The expression of Cx43 is shown in red, glial fibrillary acidic protein (GFAP) in cyan, and Cre recombinase in yellow; nuclei staining is shown in the magenta channel. Arrowheads in **(B,D)** indicate the boundary of GFAP expression and the co-localization of GFAP and Cx43 in the lateral wall of the 3V. Arrows in **(A,C)** show the expression of Cx43 in α-tanycytes and its absence or weaker expression in β-tanycytes. **(F–K)** Images of the ventricular area using the markers as described. **(J1–J3)** Images are higher magnifications of (**J**; white frames) from the dorsal to the ventral portion. Panels **(J,J1,J2)** indicate the expression of Cre recombinase in some tanycytes (arrowheads). Red arrows in the ventral region **(J,J1–J3)** point to the expression of Cre recombinase by astrocytes. 3V, third ventricle. Scale bar **(A–K)**: 100 μm. Scale bar **(J1–J3)**: 50 μm.

Taking into account that Cx30 is not expressed as high as Cx43 in tanycytes, we decided to analyze a mouse line in which the absence of Cx30 is ubiquitous, and the germ-line recombinase activity is exclusively present in GFAP-positive cells to knock out the *Cx43* gene (Wallraff et al., 2006; Zhang et al., 2013). As reported previously, Cx43^fl/fl^:hGFAP-Cre mice show a selective loss of Cx43 in astrocytes and tanycytes, but not in other Cx43-expressing cells of the brain (e.g., leptomeningeal and endothelial cells (Theis et al., 2003; Requardt et al., 2009). Cx30 expression in the brain is confined to astrocytes, tanycytes, leptomeningeal and ependymal cells (Kunzelmann et al., 1999; Glia). We first evaluated the hypothalamic localization of Cx43 in WT C57BL6J mice and compared it with the dKO line. In line with a previous study (Szilvásy-Szabó et al., 2017), Cx43 (red) was mainly localized in the wall of the 3V and in the floor of the infundibular although to a lesser degree (Figure 2A, white arrows). GFAP (cyan) is expressed by astrocytes, but it is also expressed by α-tanycytes (Figure 2B, white arrowheads), and co-localizes with Cx43 immunostaining (Figure 2C; high magnification in Figure 2D). GFAP is not expressed by β-tanycytes, which implies that it is an ideal molecule to control the expression of recombinase activity and confine it to astrocytes and α-tanycytes. Therefore, in the dKO mice (Figures 2F–K), Cx43 is lacking in astrocytes and α-tanycytes (Figures 2F–G), whereas CRE-recombinase immunostaining (yellow) co-localizes with their nuclei (magenta, Figures 2I,J), and occasionally with the GFAP marker in the parenchyma (Figure 2J, red arrow) and ventricular wall (Figure 2J, arrowheads), suggesting its expression by astrocytes and some α-tanycytes. In the dorsal (Figure 2J1), medial (Figure 2J2) and ventral (Figure 2J3) areas, immunostaining for recombinase extended further from the ventricular wall in GFAP-positive cells where Cx43 immunostaining was negative. No differences in the ventricular or parenchymal structures were detected between the wild type (Figure 2E) and dKO (Figure 2G) mice.

### Tanycytes Display Passive Membrane Current Patterns and Are Robustly Coupled With Each Other and Parenchymal Cells via Cx43

**Figure 3:**
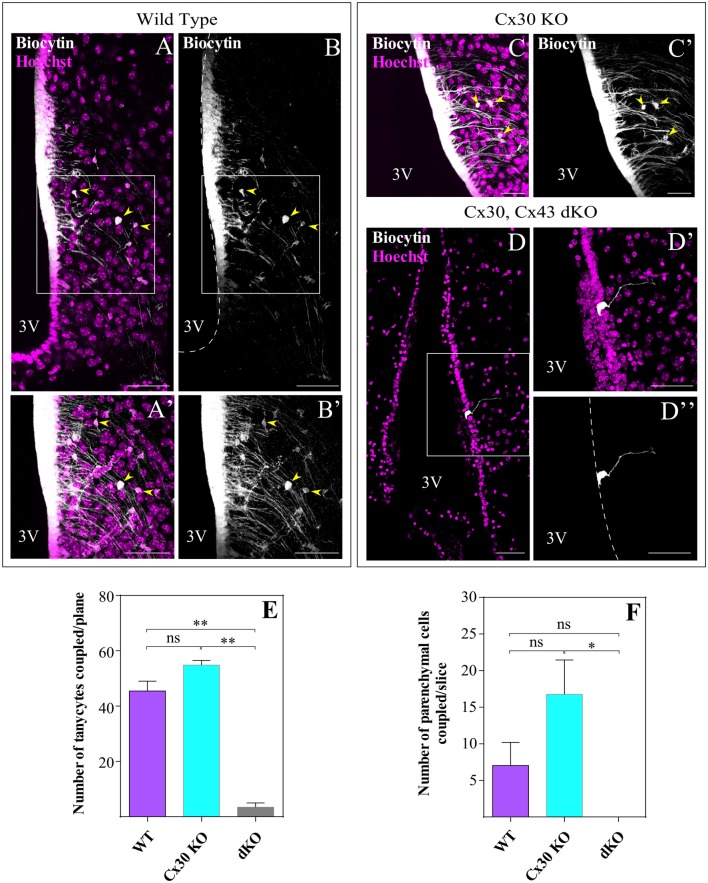
α-tanycytes are robustly coupled with each other and with parenchymal cells through Cx43. Individual tanycytes were filled with biocytin through the pipette during the patch clamp recordings. During the recording (20 min), biocytin spread through gap junctions within α-tanycytes **(A,B)** and parenchymal cells **(A′,B′)** in hypothalamic slices from WT and Cx30 KO **(C,C′)** mice. No tracer spread was observed in the dKO mice where biocytin labeling was confined to the recorded tanycyte **(D,D″)**. Panels **(A,B,D)** represent single optical planes while **(A′,B′,C,C′,D′,D″)** show the areas boxed in **(A,B,D)** at higher magnification (projection of z-stacks of five optical planes). Yellow arrows show the parenchymal cells coupled. **(E,F)** The bar graphs give the proportion of biocytin-positive tanycytes and parenchymal cells, respectively. 3V, third ventricle. Scale bar **(A–F)**: 50 μm (*N* = 5 animals over 38 days old and at least 12 slices of each genotype; data are represented as means ± SEM; *P* < 0.05; one-way ANOVA, Bonferroni *post hoc* analysis). *P < 0.05, **P < 0.01, ns, not significant.

We next evaluated coupling of α-tanycytes through gap junctions in acute frontal hypothalamic slices isolated from WT mice by filling an individual cell with biocytin, a connexin-permeable molecule of ~0.3 kDa capable of diffusing through the gap junction-coupled network, during whole-cell patch clamp recording. A single α-tanycyte was identified by its position in the ventricular wall and its polarized morphology; the cellular body faces the ventricle, and its long and unique processes extend deeply into the parenchyma. Tanycytes were held at −80 mV and filled for 20 min, checking every 10 min that the access resistance did not increase beyond 20 MΩ. While recording, de- and hyperpolarizing voltage steps (+20 mV to −160 mV) in 10 mV increments were applied in order to evoke the current pattern of tanycytes, which remained passive throughout recording (not shown). In WT littermates, biocytin injection into an α-tanycyte revealed a large coupled network of cells lining the ventricle (Figures 3A–B′, white channel) as detected by biocytin spread to (mean ± SE) 45.4 ± 3.6 cells/plane (Figure 3E), which corresponded to tanycytes judged by their typical morphology and location. Because tanycytes form an epithelium of pseudostratified appearance with nuclei that are difficult to recognize three-dimensionally, only one focal plane was analyzed. Interestingly, the superposition of different optical planes showed cells marked with biocytin in the parenchyma, which were not tanycyte processes, indicating that the coupling network extends beyond the ventricular wall (7 ± 3.2 cells/slice; *n* = 11 slices, *N* = 5 animals (Figures 3A′,B, yellow arrowheads). We next evaluated the biocytin spread in α-tanycytes of slices from either Cx30 KO or dKO mice. Similarly, when an α-tanycyte from Cx30 KO animals was filled with biocytin, the tracer spread along the ventricular wall at 54.8 ± 1.7 cells (*n* = 13 slices, *N* = 5 animals) coupled per plane (Figures 3C,C′,E), and the number of biocytin-positive cells in the parenchyma (16.8 ± 4.7 cells/slice; *n* = 13 slices, *N* = 5 animals) was not significantly different from that observed in the WT cells (Figure 3F). To examine whether Cx43 depletion is sufficient to suppress gap junction communication in α-tanycytes, we analyzed cells from dKO mice, which showed disruption of gap junctional communication (Figures 3D–D′′) in the ventricular wall with a residual coupling of 3.4 ± 1.6 cells/plane (*n* = 12 slices, *N* = 5 animals; Figure 3E) and complete disruption in parenchymal cells (*n* = 11 slices, *N* = 5 animals; Figure 3F). These results were related with the tracer spread along the ventricular wall, which corresponded to 195.7 ± 33.7 μm, 225.4 ± 56.6 μm and 16.7 ± 4.6 μm for the WT, Cx30 KO and dKO samples, respectively (Supplementary Figure S2; at least 12 slices and five animals for each genotype).

**Figure 4:**
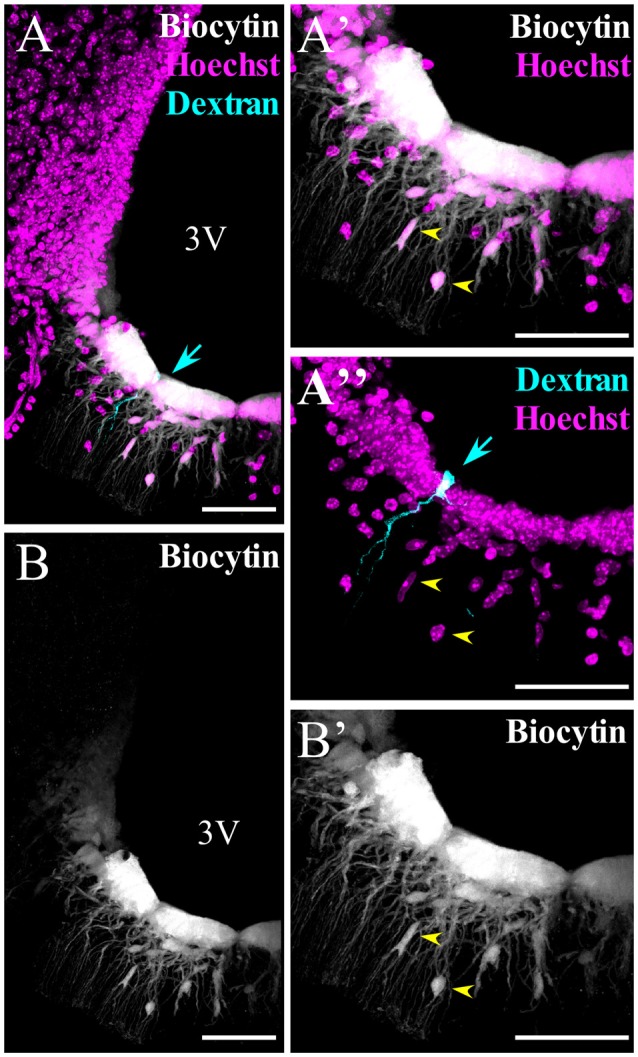
β-tanycytes are also highly coupled with each other and with parenchymal cells. **(A–B′)** Spread of biocytin through β-tanycytes and parenchymal cells. **(A′,A″,B′)** Higher magnification of the median eminence (ME) portion, where β-tanycytes localize. **(A,A″)** To identify the cell that was initially patched, it was filled with dextran Texas red (3 kDa, cyan arrow). Yellow arrows in **(A′,A″,B′)** show the parenchymal cells coupled. 3V, third ventricle. Scale bar: 50 μm.

We also analyzed if the panglial coupling detected in WT α-tanycytes exists in β-tanycytes given that they do not express GFAP (Robins et al., 2013). A single β-tanycyte was identified by the location of its cellular body on the floor of the ventricle and its prolongation that extends into the vasculature of the ME. This subpopulation of tanycytes displayed a passive current pattern in de- and hyperpolarizing voltage steps of 10 mV (+20 mV to −160 mV, holding potential −80 mV; not shown). To identify the cell that was initially patched, dextran Texas red (3 kDa) was added to the pipette (Figures 4A,A”, cyan arrow). As in α-tanycytes, β-tanycytes were also highly coupled with each other (Figures 4A–B′). Importantly, β-tanycytes showed coupling with parenchymal cells located in the ME, which by their localization and morphology could have been astrocytes or oligodendrocytes (Figures 4A′–B′).

### Characterization of the Panglial Coupling Network

**Figure 5:**
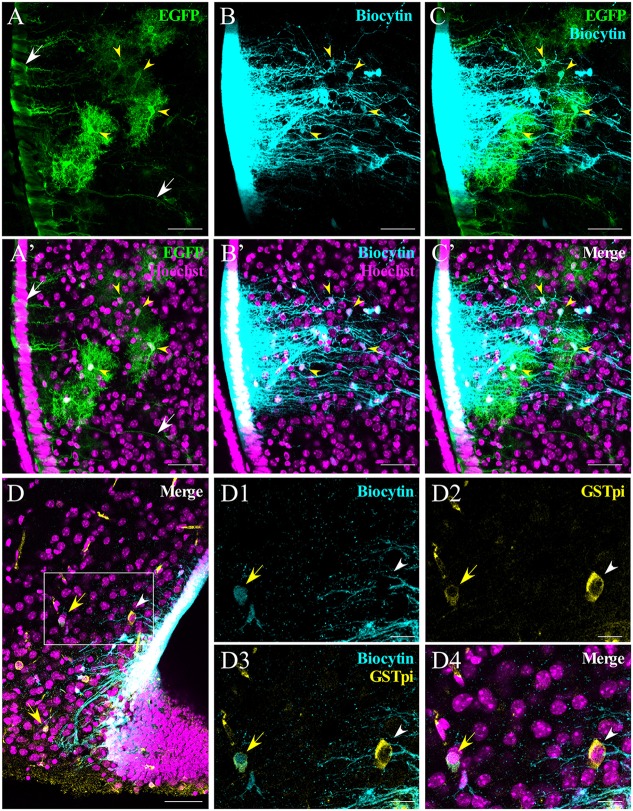
Analysis of α-tanycyte-mediated panglial coupling. α-tanycytes in slices from human GFAP-enhanced green fluorescent protein (hGFAP-EGFP) mice were filled with biocytin to evaluate panglial coupling between tanycytes and astrocytes. **(A)** Immunohistochemistry against EGFP was performed in order to enhance the signal. White arrows indicate the somata and the processes of EGFP-labeled tanycytes. **(B)** Biocytin spreads to the neighboring tanycytes and parenchymal cells that co-localize with EGFP-positive astrocytes (**C**; yellow arrowheads). **(A’–C’)** show the respective images merged with the nuclear staining (Hoechst). **(D–D4)** Occasionally (in this case, in a Cx30 KO mouse), panglial coupling of the tanycyte with oligodendrocytes was observed. **(D)** Immunoreaction against GSTpi shows oligodendrocytes close to the ventral part of the hypothalamus. The merged pictures **(D,D4)** show GSTpi (yellow), biocytin (cyan) and nuclei (Hoechst, magenta). Coupled and non-coupled oligodendrocytes are shown with a yellow arrow and white arrowhead, respectively. **(D1–D4)** Higher magnifications of **(D)**, showing biocytin in cyan **(D1)**, GSTpi in yellow **(D2)**, the merge of both channels **(D3)**, nuclei staining in magenta and the merge of all channels **(D4)**. Scale bar **(A–D)**: 50 μm; **(D1–D4)**: 20 μm.

Because the tanycyte-coupled network extended further into the hypothalamic parenchyma of WT and Cx30 KO mice (Figure 3F), we next attempted to identify the cells that were coupled to tanycytes using acute brain slices from transgenic mice that express the enhanced green fluorescent protein under the control of the human GFAP promotor; hGFAP-EGFP; P44–P64; Nolte et al., 2001). In this mouse line GFAP antibody labeling overlaps with EGFP in the majority of cells. However, some areas, such as the retina or hypothalamus, showed only low levels of EGFP expression although astrocytes were rich in GFAP (Nolte et al., 2001). Figure 5A shows that astrocytes (Figures 5A,A′, yellow arrowheads) and α-tanycytes, including their processes, were labeled (Figures 5A,A′, white arrows). After one α-tanycyte was filled with the tracer (Figures 5B,B′), we assessed the proportion of positive cells for both biocytin and GFAP in the whole slice (Figures 5C,C′), among the total amount of biocytin-positive cells, excluding ventricular cells. Quantification of α-tanycyte:astrocyte coupling showed that 11.2 ± 2.5 of a total of 36.6 ± 6.0 biocytin-positive cells/slice (13 slices from five mice) were also GFP-positive. On average, 40.5 ± 8.5% of the parenchymal cells coupled to α-tanycytes were astrocytes. In the parenchyma, coupled cells that were negative for EGFP were also detected (Figures 5C,C′). We performed immunohistochemistry with antibodies against glutathione-S-transferase Pi (GSTpi), expressed by oligodendrocytes and myelinated fibers (Tansey and Cammer, 1991) in order to determine if the oligodendrocytes were also coupled to tanycytes. We found that in some slices, biocytin (Figures 5D,D1) colocalized with the GSTpi signal (Figures 5D2–D4, yellow arrow), indicating that α-tanycytes can also establish gap junctions with some oligodendrocytes. Although the number of hypothalamic GSTpi+ cells was low, we quantified the proportion of biocytin+/GSTpi+ over the biocytin+ parenchymal cells in the hGFAP-EGFP mice (3.4% ± 2.6, *N* = 4 animals, *n* = 11 slices). Thus, tanycytes are able to establish panglial gap junction communication with astrocytes and oligodendrocytes located in the parenchyma of the hypothalamus.

### Loss of Gap Junctional Coupling Affects Transmembrane Currents in α-Tanycytes

**Figure 6:**
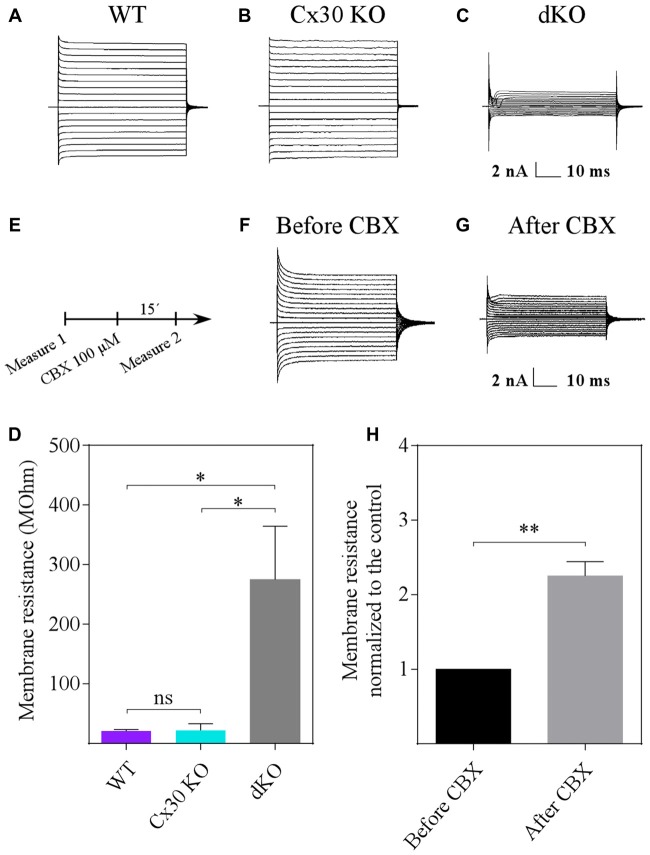
Loss of gap junction coupling affects the current pattern of α-tanycytes. **(A–D)** Compared to WT cells **(A,D)**, deletion of Cx30 did not affect the input resistance (determined at –80 to −70 mV) of α-tanycytes **(B,D)**, while deletion of Cx43 and Cx30 (dKO) led to a significant increase in the resistance **(C,D)**. **(E–H)** Decreasing gap junction coupling pharmacologically also entailed an increase in input resistance. The non-selective gap junction blocker carbenoxolone (CBX) was applied as indicated in **(E)**. After control recordings in normal artificial cerebrospinal fluid (aCSF) solution **(F,H)**, CBX (100 μM) was added (15 min), leading to a significant increase in input resistance **(G,H)** (*N* = 4 animals and 12 slices; data are represented as means ± SEM; Student’s *t*-test). *P < 0.05, **P < 0.01, ns, not significant.

We monitored electrophysiology parameters, such as resting potential and membrane resistance, of α-tanycytes within WT, Cx30 KO and dKO acute hypothalamic slices. The resting potential did not vary significantly between the three mouse lines (−74.1 ± 0.7 mV for WT (*n* = 12 slices, *N* = 5 animals), −74.7 ± 0.5 mV for Cx30 KO (*n* = 13 slices, *N* = 5 animals) and −71.2 ± 3.7 mV for dKO (*n* = 12 slices, *N* = 5 animals; data not shown). In addition, the passive whole-cell current pattern was similar between the WT and Cx30 KO samples (Figures 6A,B,D) However, significantly reduced transmembrane currents were elicited in α-tanycytes from dKO slices (Figure 6C), reflecting a significant increase in the input resistance (voltage step from −80 to −70 mV: 20.8 ± 2.5 MΩ, 22.1 ± 11.1 MΩ and 274.7 ± 89.7 MΩ for WT, Cx30 KO and dKO mice, respectively; Figure 6D; at least 12 slices and five animals for each genotype).

To confirm that loss of gap junction coupling significantly affects tanycyte transmembrane currents, the non-specific gap junction blocker, CBX was applied (Figures 6E–G). Indeed, bath application of CBX (100 μM, 15 min) led to a 2.3-fold (from 22.3 ± 5.4 to 50.5 ± 16.9 MΩ, *N* = 4 animals, *n* = 12 slices) increase in input resistance (Figure 6H). Together, these results indicate that a significant proportion of the transmembrane currents of α-tanycytes are due to gap junctions formed by Cx43.

### Loss of Gap Junction Coupling Affects Hypothalamic Basal Proliferation

**Figure 7:**
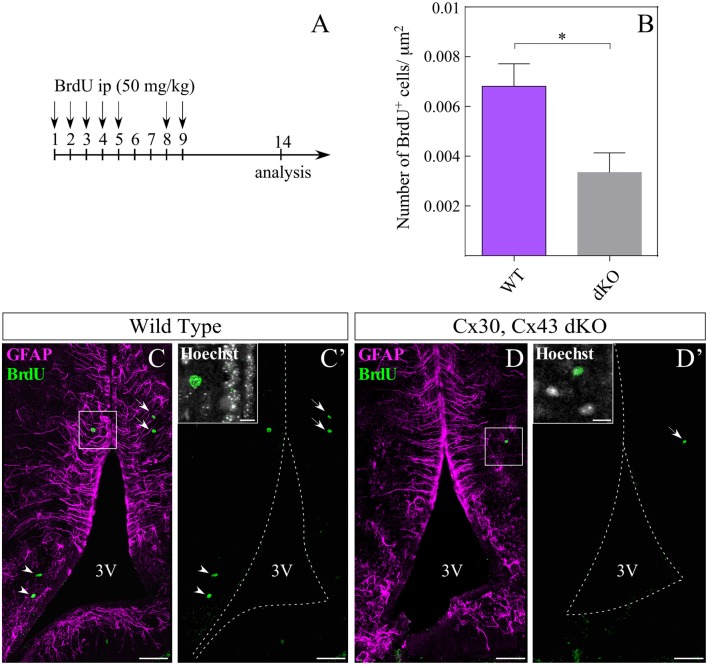
Loss of gap junction coupling affects hypothalamic cell proliferation. Intraperitoneal injection of bromodeoxyuridine (BrdU) was performed as described in **(A)**. **(B)** Number of BrdU-positive cells in the parenchyma was quantified in three dKO (13 slices) and four wild type mice (23 slices) and the results were normalized to the area analyzed (μm^2^), using the ROI manager of ImageJ software. Statistical analysis was performed using the non-parametric Mann Whitney one tail test, *P < 0.05. Representative immunohistochemistry using anti-BrdU (green) and anti-GFAP (magenta) antibodies in frontal sections of WT **(C,C′)** and dKO **(D,D′)** animals. Boxes in **(C′,D′)** indicate co-localization of anti-BrdU with nuclei staining (Hoechst). Arrowheads indicate BrdU staining close to the 3V, while arrows indicate BrdU-positive cells distal to the 3V. 3V, third ventricle. Scale bar **(C,C′,D,D′)**: 50 μm, Scale bar (Hoechst box): 10 μm.

Because tanycytes may act as adult hypothalamic precursor cells (Lee and Blackshaw, 2012; Haan et al., 2013; Robins et al., 2013; Jourdon et al., 2016), we evaluated if hypothalamic BrdU incorporation was affected by loss of gap junction coupling. WT and dKO mice were injected with BrdU as shown in Figure 7A after which BrdU-positive cells were detected in both the proximal and distal areas from the 3V (Figure 7C, arrowheads and arrows, respectively). Nuclear colocalization visualized with Hoechst staining was detected (Figures 7C′,D′, upper boxes). Immunolocalization assays showed that dKO animals had less BrdU-positive cells (Figures 7D,D′) compared to WT animals (Figures 7C,C′). Quantification of the density of BrdU-positive cells in the parenchymal area using the ROI manager of ImageJ software (Figure 7B) revealed 6.8 × 10^−3^ and 3.3 × 10^−3^ BrdU-positive cells/μm^2^) in the WT mice (*n* = 23 slices; *N* = 4 animals) and dKO animals (*n* = 13 slices; *N* = 3 animals), respectively. This suggests that loss of Cx43 significantly decreases the self-renewal capacity of hypothalamic cells.

## Discussion

In the hypothalamus, radial glia-like cells, tanycytes, may function as metabolic gatekeepers, regulating parenchymal access and availability of peripheral nutritional hormones (Langlet et al., 2013; Balland et al., 2014; Collden et al., 2015) and also acting as NPCs (Lee and Blackshaw, 2012; Haan et al., 2013; Robins et al., 2013; Jourdon et al., 2016). In the present study, transcriptional, RT-qPCR, RNA-seq analyses showed that Cx43 is the most abundantly isoform expressed by NPCs in the hypothalamus. It is important to note that Cx45, Px1 and Px2 (the last two being homologs of connexins but by now have not been shown to form gap junctions *in vivo*) are also robustly expressed in tanycytes. Although the transcriptional amount of Cx43 is almost a thousand times higher over Cx26 and Cx30 in enriched tanycyte cultures, the difference is less in whole hypothalamic extracts, likely due to the presence of other cell types. For instance, astrocytes in the hippocampus and neocortex form gap junctions mainly through Cx43, while oligodendrocytes do so through Cx32 and Cx47 (Griemsmann et al., 2015). However, in the thalamus, the majority of astrocytic gap junctions are formed by Cx30 rather than Cx43 (Griemsmann et al., 2015). These data suggest that gap junction formation varies between brain regions and cellular subpopulations. In murine hypothalamus, immunohistochemical assays showed increased Cx43 in the walls of the 3V and less reactivity in the floor of the infundibular recess, where β-tanycytes contact the ME. As different protein expression patterns have been observed among tanycyte subtypes (Campbell et al., 2017; Chen et al., 2017), it is possible that Cx43 forms the majority of gap junctions in α-tanycytes while other connexins participate in the gap junctions of β-tanycytes, which according to our RNAseq data could correspond to Cx45. In fact, using mice with genetic deletion of Cx30 and/or Cx43, we confirmed that gap junction coupling between α-tanycytes and glial cells located in the parenchyma was dependent on Cx43. It is important to note that the absence of Cx43 prevents communication between tanycytes and parenchymal cells. Nevertheless, it did not completely abolish coupling between α-tanycytes themselves since in some slices we noticed residual Spread of the tracer. One possibility is that some germline double deficient tanycytes upregulate other connexins to compensate for the lack of Cx43. For example, Cx45, which is also highly expressed in tanycytes *in vitro*, may supply some redundancy in this context. Another possibility is that the residual coupling detected in dKO mice could be attributed to the presence of Px1 (mRNA expressed highly in tanycytes) since it has been reported that Px1 and 3 can form gap junctions in cell culture (RNA). However, it has been described that unlike Px hemichannels, Px gap junctions are insensitive to CBX and probenecid (Sahu et al., 2014). In our study, we show that tanycyte coupling is sensitive to CBX. Thus, it is more likely that residual coupling in dKO mice is due to the presence of gap junctions formed by Cx45.

We then evaluated if the coupled cells in the parenchyma have a glial phenotype. To facilitate colocalization of biocytin in different cell types, we used the hGFAP-EGFP transgenic mouse line and identified that almost half of the parenchymal cells coupled to α-tanycytes were astrocytes. In transgenic hGFAP-EGFP mice, a high level of EGFP expression in astrocytes of most brain regions was observed; however, some regions, such as the retina or hypothalamus, showed only low levels of EGFP expression even though the astrocytes were rich in GFAP (Nolte et al., 2001). Therefore, the number of astrocytes coupled to tanycytes reported here is likely to be underestimated. Importantly, astrocytes as well as oligodendrocytes are coupled to tanycytes.

Panglial-coupled networks were previously characterized in other brain areas, such as the thalamus, hippocampus, neocortex (Wasseff and Scherer, 2011; Griemsmann et al., 2015) and corpus callosum (Maglione et al., 2010; Meyer et al., 2018). In the different brain areas, the proportion of oligodendrocytes/astrocytes per network and the expression of connexin isoforms differ (Griemsmann et al., 2015). However, the physiological impact of the hypothalamic panglial coupling network identified in the present study remains to be elucidated.

In the corpus callosum, a recently described panglial network cooperates to transfer glucose and lactate via GLUT1 and monocarboxylate transporter 1 (MCT1) to sustain metabolic support to axons (Meyer et al., 2018). As part of the glucosensing mechanism, tanycytes express GLUT2 and MCT1 and MCT4 (García et al., 2003; Elizondo-Vega et al., 2016). Their individual inhibition *in vivo* is sufficient to disrupt the communication between the metabolic state of the organism and the response of anorexigenic/orexigenic neurons, thus altering feeding behavior (Elizondo-Vega et al., 2016; Barahona et al., 2018). It is unknown whether the panglial network discovered here is an additional path to transmit metabolites (originating from the CSF or fenestrated capillaries of the ME) to neurons of adjacent nuclei. Moreover, the coupled network of tanycytes and astrocytes could function as a glial syncytium to coordinate and amplify the information coming from both CSF (through tanycytes) and peripheral circulation (through astrocytes), thereby transmitting a consensus response despite the structural limitations imposed by the blood-brain barrier. The structural barrier formed by tight junctions of β-tanycytes between the CSF and ME can be modified by the metabolic state (Langlet et al., 2013). Thus, it is likely that the metabolic conditions also affect the extension of the coupled network. Indeed, changes in metabolic status e.g., fasting, alter the expression of mediobasal hypothalamic Cx43, but not Cx30 levels (Allard et al., 2014). Moreover, transient downregulation of Cx43 in the mediobasal hypothalamus disrupts the insulinemia response to intracarotid glucose injection (Allard et al., 2014). This fact exemplifies the requirement of connexins for hypothalamic glucose detection.

Previous studies found that Lucifer Yellow easily spread through α- and β-tanycyte gap junctions; however, these studies did not detect panglial coupling (Jarvis and Andrew, 1988; Szilvásy-Szabó et al., 2017). Instead, coupling to hypophysiotropic neuronal axons were mentioned. The results obtained in this study cannot exclude the possibility that the coupled network extends further than to astrocytes and oligodendrocytes, but a potential coupling to other cell types remains to be elucidated. Although all connexins are permeable to small molecules, Lucifer Yellow and biocytin (the two most often used tracers to measure coupling) differ in their gap junction permeability. For instance, the positively charged neurobiotin is able to pass through gap junctions formed by Cx30, while the negatively charged Lucifer yellow does not (Yum et al., 2007). Therefore, differences in the net charge of both tracers could explain in part why the previous studies mentioned above did not find panglial coupling.

All the cells investigated here displayed passive currents when applying hyper- and depolarization voltage steps. Genetic deletion of connexins or pharmacological inhibition of gap junction coupling was associated with a drastic increase in membrane resistance, confirming previous findings (Blomstrand et al., 2004; Wallraff et al., 2006; Russo et al., 2008). Interestingly CBX treatment increased the input resistance of tanycyte membranes by 2-fold, similar to what has been observed in hippocampal astrocytes (Blomstrand et al., 2004). This increase was less as compared to the changes seen in cells from dKO mice, which might indicate an insufficient CBX-mediated block. Alternatively, it is possible that permanent lack of the *Cx43* gene in dKO mice led to compensatory alterations in the expression of other transmembrane channels.

In adult WT animals, BrdU analysis showed few if any proliferative tanycytes, which correlates with a low rate of tanycyte self-renewal previously reported (Hendrickson et al., 2018) and low hypothalamic neurogenesis rate compared to other neurogenic niches (Lledo et al., 2006; Migaud et al., 2010). However, the protocol used here to detect the proliferative state of hypothalamic cells included a rest week after BrdU injections, leaving enough time for ventricular precursor cells to migrate into the parenchyma. Although the fate of proliferative cells was not elucidated in this study, it is likely that the lack of Cx43 affects not only tanycyte coupling but also their self-renewal ability, among other cellular functions. Additionally, it is not possible to rule out that Cx43 could have effects on proliferation that do not involve cell coupling. For instance, BrdU labeling demonstrated that the loss of Cx43 impairs hippocampal neurogenesis (Kunze et al., 2009). Finally, the physiological role of tanycytic Cx43 in forming communicating junctions between tanycytes and glial cells located close to the arcuate nucleus may include cell cycle regulation of NPCs through the spread of calcium waves (Weissman et al., 2004; Frayling et al., 2011), metabolic signal transmission to hypothalamic neurons, buffering of ions and homeostatic regulation of metabolites present in the CSF and in the peripheral blood. Thus, the cellular network coupled by Cx43-gap junctions might regulate short- and long-term hypothalamic function.

## Author Contributions

The experiments were performed at the Department of Cell Biology at the University of Concepcion and in the Institute of Cellular Neurosciences, University of Bonn. AR, MG-R, CS and TC conceived the experiments. AR, CS and MG-R designed the experiments. AR, AT, GS, RE-V, AP, CP, ET-S, MS and SL performed the experiments. AR, ET-S, TC and MS analyzed the data. MG-R, CS, TC and AR contributed to reagents, materials and analysis tools. AR, CS, TC and MG-R wrote the article. RE-V, ET-S, GS, CS and CP critically revised the manuscript. All authors have approved the final version of the manuscript and agree to be accountable for all aspects of the work in ensuring that questions related to the accuracy or integrity of any part of the work are appropriately investigated and resolved. All persons designated as authors qualify for authorship, and all those who qualify for authorship are listed.

## Conflict of Interest Statement

The authors declare that the research was conducted in the absence of any commercial or financial relationships that could be construed as a potential conflict of interest.

## Supplementary Material

FIGURE S1 Connexins (Cxs) and Pannexins (Panx) expression. **(A,B)** Immunohistochemistry of a coronal mouse hypothalamic section (P46) using antibodies to detect Cx45 (white channel) and Vimentin (yellow channel). TOPRO nuclei staining is shown in magenta. **(C–J)**. High magnification (60×) of **(A,B)** boxes showing Cx45 expression in α- **(C–F)** and β-tanycytes **(G–J)**. 3V, Third ventricle. ME, Median Eminence. Scale bar: 50 μm. **(K)** Expression of Cx43, Cx45, Panx1 and Panx2 was measured by RT-qPCR in primary cultures of rat tanycytes (P1, *N* = 3, 4, 5 and 5 independent primary cultures for Cx43, Cx45, Panx1 and Panx2, respectively). Transcriptional expression was normalized to the cyclophilin housekeeping gene.

FIGURE S2 Length of biocytin spread and membrane potential of WT, Cx30 KO and dKO α-tanycytes. **(A)** Spread of biocytin along the lateral wall measured in micrometers (μm). **(B)** No significant differences were observed between the wild type (−74.1 ± 0.7 mV), Cx30 KO (−74.7 ± 0.5 mV) and dKO (−71.2 ± 3.7 mV) α-tanycyte membrane potential. (*N* = 5 animals over 38 days old and at least 12 slices of each genotype; data are represented as means ± SEM; *P* < 0.05; one-way ANOVA, Bonferroni *post hoc* analysis). **P* < 0.05, ns, not significant.

## References

[B1] Allard C. Carneiro L. Grall S. Cline B. H. Fioramonti X. Chrétien C.. (2014). Hypothalamic astroglial connexins are required for brain glucose sensing-induced insulin secretion. J. Cereb. Blood Flow Metab. 34, 339–346. 10.1038/jcbfm.2013.206 24301293 PMC3915215

[B2] Anders S. Pyl P. T. Huber W. (2015). HTSeq—a Python framework to work with high-throughput sequencing data. Bioinformatics 31, 166–169. 10.1093/bioinformatics/btu638 25260700 PMC4287950

[B3] Balland E. Dam J. Langlet F. Caron E. Steculorum S. Messina A.. (2014). Hypothalamic tanycytes are an ERK-gated conduit for leptin into the brain. Cell Metab. 19, 293–301. 10.1016/j.cmet.2013.12.015 24506870 PMC3936883

[B4] Barahona M. J. Llanos P. Recabal A. Escobar-Acuña K. Elizondo-Vega R. Salgado M.. (2018). Glial hypothalamic inhibition of GLUT2 expression alters satiety, impacting eating behavior. Glia 66, 592–605. 10.1002/glia.23267 29178321 PMC5814884

[B6] Bittman K. S. LoTurco J. J. (1999). Differential regulation of connexin 26 and 43 in murine neocortical precursors. Cereb. Cortex 9, 188–195. 10.1093/cercor/9.2.188 10220231

[B5] Bittman K. Owens D. F. Kriegstein A. R. LoTurco J. J. (1997). Cell coupling and uncoupling in the ventricular zone of developing neocortex. J. Neurosci. 17, 7037–7044. 10.1523/jneurosci.17-18-07037.1997 9278539 PMC6573271

[B7] Blomstrand F. Venance L. Sirén A. L. Ezan P. Hanse E. Glowinski J.. (2004). Endothelins regulate astrocyte gap junctions in rat hippocampal slices. Eur. J. Neurosci. 19, 1005–1015. 10.1111/j.0953-816x.2004.03197.x 15009148

[B8] Campbell J. N. Macosko E. Z. Fenselau H. Pers T. H. Lyubetskaya A. Tenen D.. (2017). A molecular census of arcuate hypothalamus and median eminence cell types. Nat. Neurosci. 20, 484–496. 10.1038/nn.4495 28166221 PMC5323293

[B9] Chen R. Wu X. Jiang L. Zhang Y. (2017). Single-cell RNA-Seq reveals hypothalamic cell diversity. Cell Rep. 18, 3227–3241. 10.1016/j.celrep.2017.03.004 28355573 PMC5782816

[B10] Collden G. Balland E. Parkash J. Caron E. Langlet F. Prevot V.. (2015). Neonatal overnutrition causes early alterations in the central response to peripheral ghrelin. Mol. Metab. 4, 15–24. 10.1016/j.molmet.2014.10.003 25685686 PMC4314535

[B11] Córtés-Campos C. Elizondo R. Llanos P. Uranga R. M. Nualart F. García M. A. (2011). MCT expression and lactate influx/efflux in tanycytes involved in glia-neuron metabolic interaction. PLoS One 6:e16411. 10.1371/journal.pone.0016411 21297988 PMC3030577

[B12] Elizondo-Vega R. Córtés-Campos C. Barahona M. J. Carril C. Ordenes P. Salgado M.. (2016). Inhibition of hypothalamic MCT1 expression increases food intake and alters orexigenic and anorexigenic neuropeptide expression. Sci. Rep. 6:33606. 10.1038/srep33606 27677351 PMC5039692

[B13] Elizondo-Vega R. Córtes-Campos C. Barahona M. J. Oyarce K. A. Carril C. A. García-Robles M. A. (2015). The role of tanycytes in hypothalamic glucosensing. J. Cell. Mol. Med. 19, 1471–1482. 10.1111/jcmm.12590 26081217 PMC4511346

[B14] Frayling C. Britton R. Dale N. (2011). ATP-mediated glucosensing by hypothalamic tanycytes. J. Physiol. 589, 2275–2286. 10.1113/jphysiol.2010.202051 21486800 PMC3098703

[B15] Freitas A. S. Xavier A. L. Furtado C. M. Hedin-Pereira C. Fróes M. M. Menezes J. R. (2012). Dye coupling and connexin expression by cortical radial glia in the early postnatal subventricular zone. Dev. Neurobiol. 72, 1482–1497. 10.1002/dneu.22005 22234946

[B16] García M. A. Millán C. Balmaceda-Aguilera C. Castro T. Pastor P. Montecinos H.. (2003). Hypothalamic ependymal-glial cells express the glucose transporter GLUT2, a protein involved in glucose sensing. J. Neurochem. 86, 709–724. 10.1046/j.1471-4159.2003.01892.x 12859684

[B17] Gouazé A. Brenachot X. Rigault C. Krezymon A. Rauch C. Nédélec E.. (2013). Cerebral cell renewal in adult mice controls the onset of obesity. PLoS One 8:e72029. 10.1371/journal.pone.0072029 23967273 PMC3742483

[B18] Griemsmann S. Höft S. P. Bedner P. Zhang J. von Staden E. Beinhauer A.. (2015). Characterization of panglial gap junction networks in the thalamus, neocortex and hippocampus reveals a unique population of glial cells. Cereb. Cortex 25, 3420–3433. 10.1093/cercor/bhu157 25037920 PMC4585496

[B19] Haan N. Goodman T. Najdi-Samiei A. Stratford C. M. Rice R. El Agha E.. (2013). Fgf10-expressing tanycytes add new neurons to the appetite/energy-balance regulating centers of the postnatal and adult hypothalamus. J. Neurosci. 33, 6170–6180. 10.1523/jneurosci.2437-12.2013 23554498 PMC3736310

[B20] Hendrickson M. L. Zutshi I. Wield A. Kalil R. E. (2018). Nestin expression and *in vivo* proliferative potential of tanycytes and ependymal cells lining the walls of the third ventricle in the adult rat brain. Eur. J. Neurosci. 47, 284–293. 10.1111/ejn.13834 29359828

[B21] Jarvis C. R. Andrew R. D. (1988). Correlated electrophysiology and morphology of the ependyma in rat hypothalamus. J. Neurosci. 8, 3691–3702. 10.1523/jneurosci.08-10-03691.1988 3193176 PMC6569601

[B22] Jourdon A. Gresset A. Spassky N. Charnay P. Topilko P. Santos R. (2016). Prss56, a novel marker of adult neurogenesis in the mouse brain. Brain Struct. Funct. 221, 4411–4427. 10.1007/s00429-015-1171-z 26701169

[B23] Kokoeva M. V. Yin H. Flier J. S. (2005). Neurogenesis in the hypothalamus of adult mice: potential role in energy balance. Science 310, 679–683. 10.1126/science.1115360 16254185

[B24] Kunze A. Congreso M. R. Hartmann C. Wallraff-Beck A. Hüttmann K. Bedner P.. (2009). Connexin expression by radial glia-like cells is required for neurogenesis in the adult dentate gyrus. Proc. Natl. Acad. Sci. U S A 106, 11336–11341. 10.1073/pnas.0813160106 19549869 PMC2700144

[B25] Kunzelmann P. Schröder W. Traub O. Steinhäuser C. Dermietzel R. Willecke K. (1999). Late onset and increasing expression of the gap junction protein connexin30 in adult murine brain and long-term cultured astrocytes. Glia 25, 111–119. 10.1002/(sici)1098-1136(19990115)25:2<111::aid-glia2>3.0.co;2-i 9890626

[B26] Lacar B. Young S. Z. Platel J. C. Bordey A. (2011). Gap junction-mediated calcium waves define communication networks among murine postnatal neural progenitor cells. Eur. J. Neurosci. 34, 1895–1905. 10.1111/j.1460-9568.2011.07901.x 22098557 PMC3237798

[B27] Langlet F. Mullier A. Bouret S. G. Prevot V. Dehouck B. (2013). Tanycyte-like cells form a blood-cerebrospinal fluid barrier in the circumventricular organs of the mouse brain. J. Comp. Neurol. 521, 3389–3405. 10.1002/cne.23445 23649873 PMC3973970

[B28] Lee D. A. Blackshaw S. (2012). Functional implications of hypothalamic neurogenesis in the adult mammalian brain. Int. J. Dev. Neurosci. 30, 615–621. 10.1016/j.ijdevneu.2012.07.003 22867732 PMC3906127

[B29] Liebmann M. Stahr A. Guenther M. Witte O. W. Frahm C. (2013). Astrocytic Cx43 and Cx30 differentially modulate adult neurogenesis in mice. Neurosci. Lett. 545, 40–45. 10.1016/j.neulet.2013.04.013 23618652

[B30] Lledo P. M. Alonso M. Grubb M. S. (2006). Adult neurogenesis and functional plasticity in neuronal circuits. Nat. Rev. Neurosci. 7, 179–193. 10.1038/nrn1867 16495940

[B31] Maglione M. Tress O. Haas B. Karram K. Trotter J. Willecke K.. (2010). Oligodendrocytes in mouse corpus callosum are coupled via gap junction channels formed by connexin47 and connexin32. Glia 58, 1104–1117. 10.1002/glia.20991 20468052

[B32] Meyer N. Richter N. Fan Z. Siemonsmeier G. Pivneva T. Jordan P.. (2018). Oligodendrocytes in the mouse corpus callosum maintain axonal function by delivery of glucose. Cell Rep. 22, 2383–2394. 10.1016/j.celrep.2018.02.022 29490274

[B33] Migaud M. Batailler M. Segura S. Duittoz A. Franceschini I. Pillon D. (2010). Emerging new sites for adult neurogenesis in the mammalian brain: a comparative study between the hypothalamus and the classical neurogenic zones. Eur. J. Neurosci. 32, 2042–2052. 10.1111/j.1460-9568.2010.07521.x 21143659

[B34] Nagy J. I. Rash J. E. (2000). Connexins and gap junctions of astrocytes and oligodendrocytes in the CNS. Brain Res. Rev. 32, 29–44. 10.1016/s0165-0173(99)00066-1 10751655

[B35] Nolte C. Matyash M. Pivneva T. Schipke C. G. Ohlemeyer C. Hanisch U. K.. (2001). GFAP promoter-controlled EGFP-expressing transgenic mice: a tool to visualize astrocytes and astrogliosis in living brain tissue. Glia 33, 72–86. 10.1002/1098-1136(20010101)33:1<72::AID-GLIA1007>3.0.CO;2-A 11169793

[B36] Orellana J. A. Sáez P. J. Córtés-Campos C. Elizondo R. J. Shoji K. F. Contreras-Duarte S.. (2012). Glucose increases intracellular free Ca^2+^ in tanycytes via ATP released through connexin 43 hemichannels. Glia 60, 53–68. 10.1002/glia.21246 21987367 PMC3417330

[B37] Pencea V. Bingaman K. D. Wiegand S. J. Luskin M. B. (2001). Infusion of brain-derived neurotrophic factor into the lateral ventricle of the adult rat leads to new neurons in the parenchyma of the striatum, septum, thalamus and hypothalamus. J. Neurosci. 21, 6706–6717. 10.1523/jneurosci.21-17-06706.2001 11517260 PMC6763082

[B38] Pérez-Martín M. Cifuentes M. Grondona J. M. López-Avalos M. D. Gómez-Pinedo U. García-Verdugo J. M.. (2010). IGF-I stimulates neurogenesis in the hypothalamus of adult rats. Eur. J. Neurosci. 31, 1533–1548. 10.1111/j.1460-9568.2010.07220.x 20525067

[B39] Requardt R. P. Kaczmarczyk L. Dublin P. Wallraff-Beck A. Mikeska T. Degen J.. (2009). Quality control of astrocyte-directed Cre transgenic mice: the benefits of a direct link between loss of gene expression and reporter activation. Glia 57, 680–692. 10.1002/glia.20796 18942753

[B40] Robins S. C. Stewart I. McNay D. E. Taylor V. Giachino C. Goetz M.. (2013). α-Tanycytes of the adult hypothalamic third ventricle include distinct populations of FGF-responsive neural progenitors. Nat. Commun. 4:2049. 10.1038/ncomms3049 23804023

[B41] Rodríguez E. M. Blázquez J. L. Pastor F. E. Peláez B. Peña P. Peruzzo B.. (2005). Hypothalamic tanycytes: a key component of brain-endocrine interaction. Int. Rev. Cytol. 247, 89–164. 10.1016/s0074-7696(05)47003-5 16344112

[B42] Russo R. E. Reali C. Radmilovich M. Fernández A. Trujillo-Cenóz O. (2008). Connexin 43 delimits functional domains of neurogenic precursors in the spinal cord. J. Neurosci. 28, 3298–3309. 10.1523/jneurosci.5736-07.2008 18367597 PMC6670595

[B43] Sahu G. Sukumaran S. Bera A. K. (2014). Pannexins form gap junctions with electrophysiological and pharmacological properties distinct from connexins. Sci. Rep. 4:4955. 10.1038/srep04955 24828343 PMC4021813

[B44] Salgado M. Tarifeño-Saldivia E. Ordenes P. Millán C. Yañez M. J. Llanos P.. (2014). Dynamic localization of glucokinase and its regulatory protein in hypothalamic tanycytes. PLoS One 9:e94035. 10.1371/journal.pone.0094035 24739934 PMC3989220

[B45] Szilvásy-Szabó A. Varga E. Beliczai Z. Lechan R. M. Fekete C. (2017). Localization of connexin 43 gap junctions and hemichannels in tanycytes of adult mice. Brain Res. 1673, 64–71. 10.1016/j.brainres.2017.08.010 28803831

[B46] Talaverón R. Fernández P. Escamilla R. Pastor A. M. Matarredona E. R. Sáez J. C. (2015). Neural progenitor cells isolated from the subventricular zone present hemichannel activity and form functional gap junctions with glial cells. Front. Cell. Neurosci. 9:411. 10.3389/fncel.2015.00411 26528139 PMC4602088

[B47] Tansey F. A. Cammer W. (1991). A pi form of glutathione-S-transferase is a myelin- and oligodendrocyte-associated enzyme in mouse brain. J. Neurochem. 57, 95–102. 10.1111/j.1471-4159.1991.tb02104.x 1711102

[B48] Theis M. Jauch R. Zhuo L. Speidel D. Wallraff A. Döring B.. (2003). Accelerated hippocampal spreading depression and enhanced locomotory activity in mice with astrocyte-directed inactivation of connexin43. J. Neurosci. 23, 766–776. 10.1523/jneurosci.23-03-00766.2003 12574405 PMC6741919

[B49] Trapnell C. Roberts A. Goff L. Pertea G. Kim D. Kelley D. R.. (2012). Differential gene and transcript expression analysis of RNA-seq experiments with TopHat and Cufflinks. Nat. Protoc. 7, 562–578. 10.1038/nprot.2012.016 22383036 PMC3334321

[B50] Wallraff A. Köhling R. Heinemann U. Theis M. Willecke K. Steinhäuser C. (2006). The impact of astrocytic gap junctional coupling on potassium buffering in the hippocampus. J. Neurosci. 26, 5438–5447. 10.1523/jneurosci.0037-06.2006 16707796 PMC6675300

[B51] Wasseff S. K. Scherer S. S. (2011). Cx32 and Cx47 mediate oligodendrocyte:astrocyte and oligodendrocyte:oligodendrocyte gap junction coupling. Neurobiol. Dis. 42, 506–513. 10.1016/j.nbd.2011.03.003 21396451 PMC3773476

[B52] Weissman T. A. Riquelme P. A. Ivic L. Flint A. C. Kriegstein A. R. (2004). Calcium waves propagate through radial glial cells and modulate proliferation in the developing neocortex. Neuron 43, 647–661. 10.1016/j.neuron.2004.08.015 15339647

[B53] Yulyaningsih E. Rudenko I. A. Valdearcos M. Dahlén E. Vagena E. Chan A.. (2017). Acute lesioning and rapid repair of hypothalamic neurons outside the blood-brain barrier. Cell Rep. 19, 2257–2271. 10.1016/j.celrep.2017.05.060 28614713 PMC5651178

[B54] Yum S. W. Zhang J. Valiunas V. Kanaporis G. Brink P. R. White T. W.. (2007). Human connexin26 and connexin30 form functional heteromeric and heterotypic channels. Am. J. Physiol. Cell Physiol. 293, C1032–C1048. 10.1152/ajpcell.00011.2007 17615163

[B55] Zhang J. Dublin P. Griemsmann S. Klein A. Brehm R. Bedner P.. (2013). Germ-line recombination activity of the widely used hGFAP-Cre and nestin-Cre transgenes. PLoS One 8:e82818. 10.1371/journal.pone.0082818 24349371 PMC3857304

