# Supplementary figures and images for: Connexin-43 Gap Junctions Are Responsible for the Hypothalamic Tanycyte-Coupled Network
Source: Front Cell Neurosci. 2018 Nov 26;12:406. doi: 10.3389/fncel.2018.00406 (PMC6275304; doi:10.3389/fncel.2018.00406)

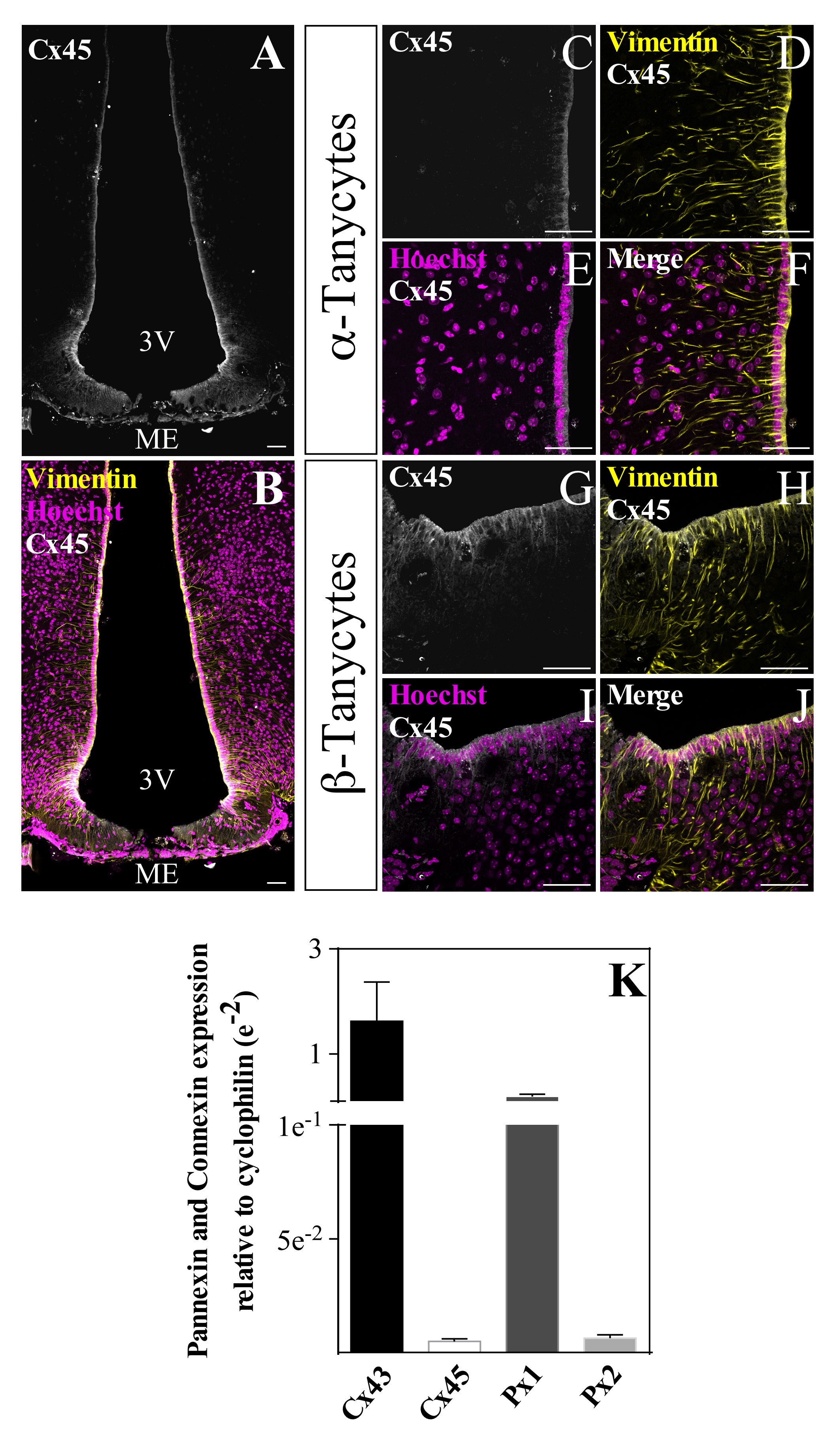

Supplement: FIGURE S1 — Connexins (Cxs) and Pannexins (Panx) expression. (A,B) Immunohistochemistry of a coronal mouse hypothalamic section (P46) using antibodies to detect Cx45 (white channel) and Vimentin (yellow channel). TOPRO nuclei staining is shown in magenta. (C–J). High magnification (60×) of (A,B) boxes showing Cx45 expression in α- (C–F) and β-tanycytes (G–J). 3V, Third ventricle. ME, Median Eminence. Scale bar: 50 μm. (K) Expression of Cx43, Cx45, Panx1 and Panx2 was measured by RT-qPCR in primary cultures of rat tanycytes (P1, N = 3, 4, 5 and 5 independent primary cultures for Cx43, Cx45, Panx1 and Panx2, respectively). Transcriptional expression was normalized to the cyclophilin housekeeping gene. [file Image_1.TIF]

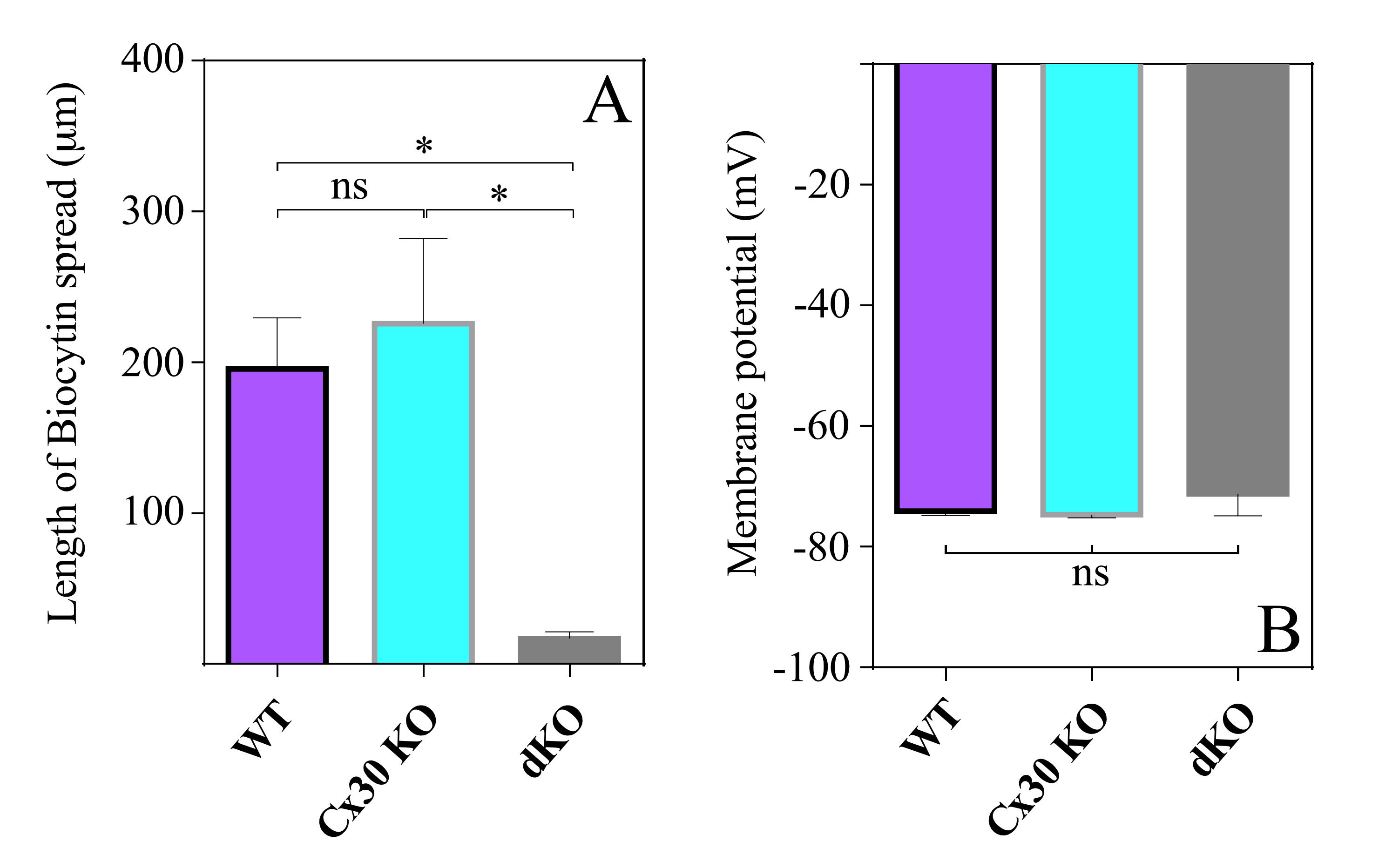

Supplement: FIGURE S2 — Length of biocytin spread and membrane potential of WT, Cx30 KO and dKO α-tanycytes. (A) Spread of biocytin along the lateral wall measured in micrometers (μm). (B) No significant differences were observed between the wild type (−74.1 ± 0.7 mV), Cx30 KO (−74.7 ± 0.5 mV) and dKO (−71.2 ± 3.7 mV) α-tanycyte membrane potential. (N = 5 animals over 38 days old and at least 12 slices of each genotype; data are represented as means ± SEM; P < 0.05; one-way ANOVA, Bonferroni post hoc analysis). *P < 0.05, ns, not significant. [file Image_2.TIF]
